# KRAS-related long noncoding RNAs in human cancers

**DOI:** 10.1038/s41417-021-00381-x

**Published:** 2021-09-06

**Authors:** Mahsa Saliani, Amin Mirzaiebadizi, Ali Javadmanesh, Akram Siavoshi, Mohammad Reza Ahmadian

**Affiliations:** 1 Institute of Biochemistry and Molecular Biology II, Medical Faculty, Heinrich Heine University, Düsseldorf, Germany; 2 Department of Chemistry, Faculty of Science, Ferdowsi University of Mashhad, Mashhad, Iran; 3 Department of Animal Science, Faculty of Agriculture, Ferdowsi University of Mashhad, Mashhad, Iran; 4 Stem Cell Biology and Regenerative Medicine Research Group, Research Institute of Biotechnology, Ferdowsi University of Mashhad, Mashhad, 9177948974 Iran; 5 Department of Biology, Sinabioelixir Group, Alborz Health Technology Development Center, Tehran, Iran

**Keywords:** Cell biology, Drug development

## Abstract

KRAS is one of the most widely prevalent proto-oncogenes in human cancers. The constitutively active KRAS oncoprotein contributes to both tumor onset and cancer development by promoting cell proliferation and anchorage-independent growth in a MAPK pathway-dependent manner. The expression of microRNAs (miRNAs) and the KRAS oncogene are known to be dysregulated in various cancers, while long noncoding RNAs (lncRNAs) can act as regulators of the miRNAs targeting *KRAS* oncogene in different cancers and have gradually become a focus of research in recent years. In this review article, we summarize recent advances in the research on lncRNAs that have sponging effects on KRAS-targeting miRNAs as crucial mediators of KRAS expression in different cell types and organs. A deeper understanding of lncRNA function in KRAS-driven cancers is of major fundamental importance and will provide a valuable clinical tool for the diagnosis, prognosis, and eventual treatment of cancers.

## Introduction

KRAS is a small GDP/GTP-binding protein that transduces extracellular signals and induces intracellular responses. KRAS cycles between an inactive, GDP-bound (“off”) state, and an active, GTP-bound (“on”) state. This off/on cycle is based on GDP/GTP exchange and GTP hydrolysis reactions stimulated by RAS-specific guanine nucleotide exchange factors (GEFs) and GTPase-activating proteins (GAPs), respectively [1]. GTP-bound KRAS transduces signals to its downstream effectors and thus activates multiple signaling pathways [2, 3]. Therefore, activated KRAS controls various cellular processes, including survival, growth, proliferation, differentiation, and apoptosis, all of which are known as hallmarks of cancer [4]. Somatic mutations in KRAS trigger the robust gain-of-function effects of oncogenic KRAS and neoplastic signal transduction owing to the reduction in GTP hydrolysis and resistance to GAP function [5, 6].

The *KRAS* oncogene has been extensively studied in human tumor malignancies [7, 8]. Intensive efforts to understand the mechanisms underlying the intracellular trafficking, regulation, and signaling pathways of KRAS have suggested several therapeutic strategies [9]. Despite its well-recognized importance in cancer promotion, only a few efforts in the past four decades have resulted in approved clinical therapeutic strategies for *KRAS*-mutant cancers [9–11]. Additionally, *KRAS* mutation is an important predictive marker in determining resistance to EGFR-targeted therapies [12]. Thus, further studies are needed to elucidate the mechanisms responsible for the modulation of *KRAS* to evaluate other potential therapeutic approaches.

**Fig. 1:**
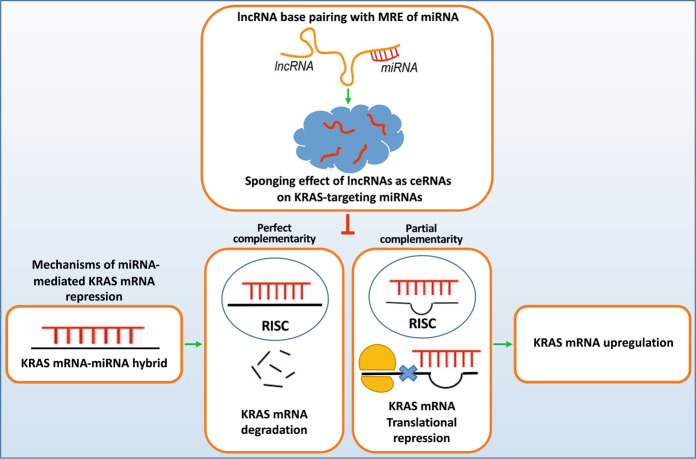
Mechanism of KRAS gene regulation by oncogenic lncRNAs through sponging effects. As key gene regulators, tumor suppressor miRNAs bind to their targets and interfere with translation. The RNA‐induced silencing complex (RISK) guides the antisense strand of the miRNA to bind to its target KRAS mRNA sequence in a complementary manner, forming a double‐stranded helix. Perfect complementarity results in endonucleolytic cleavage, while partial complementarity subjects mRNA to translational repression. Oncogenic lncRNAs act as ceRNA decoys by presenting complementary sequences with MREs to sponge miRNAs from their target KRAS mRNAs. lncRNAs consequently promote KRAS mRNA stabilization and thus its upregulation.

Long noncoding RNAs (lncRNAs) are a class of noncoding RNAs (ncRNAs) with a minimum length of 200 nucleotides, which have been well studied in the context of RNA-based therapeutics [13, 14]. Although only a small fraction of known lncRNAs have been functionally characterized, there is growing evidence of their involvement in a variety of biological processes, human diseases, and malignancies [15]. These molecules, as the key regulators of gene expression, play essential roles in a wide variety of biological processes and signaling pathways involved in the progression of many human cancers [16–19]. Emerging evidence has suggested that various lncRNAs are likely to function as competing endogenous RNAs (ceRNAs). These lncRNAs act as oncogenes by sponging tumor suppressor microRNAs (miRNAs) [20, 21], thereby indirectly regulating the expression of the genes targeted by these miRNAs [22] (Fig. 1). Considering the wide diversity of miRNAs and their high capacity for regulating hundreds of genes, many driver oncogenes, such as *ERBB2*, *BRAF*, *EGFR*, *MYC*, *SRC*, and *BCL2*, are targeted by miRNAs [23–25]. In this regard, many tumor suppressor miRNAs have inhibitory effects on *KRAS*-associated tumrigenesis by downregulating *KRAS* expression [26, 27]. Therefore, oncogenic lncRNAs, as sponges of tumor suppressor miRNAs that target *KRAS*, promote cancer development via the upregulation of the *KRAS* oncogene [28–30].

It is evident that ceRNAs and miRNA response elements (MREs) are two essential components of the ‘sponge effect´ [31]. MREs are seed regions of 2-8 nucleotides in the 5’ region of miRNA [32]. The ability of a miRNA to bind to its mRNA target and lncRNA via its MRE provides competition between mRNA and lncRNA for interaction with their target miRNA. The binding of lncRNA to miRNA as a ceRNA prevents the latter from recognizing mRNA and consequently results in its silencing. This interaction leads to the regulation of MREs on the targets, which plays an important role in posttranscriptional regulation and is known as the sponging effect [31] (Fig. 1).

Identification of mechanisms involved in *KRAS* regulation by lncRNAs is expected to greatly enhance our understanding of the mechanisms of tumorigenesis associated with *KRAS* regulation. While the sponging effect of lncRNAs on miRNAs that target *KRAS* seems to be one of the key mechanisms by which *KRAS* is regulated, details of other regulatory mechanisms remain to be elucidated. The association of lncRNAs with various regulatory apparatuses, such as chromatin remodeling factors, transcription factors, splicing machinery, nuclear trafficking modulators, and miRNAs, shows the complexity of their regulatory approaches [33, 34]. Therefore, to understand other regulatory effects of lncRNAs on *KRAS* expression, the role of all interactions between lncRNAs and other macromolecules, such as DNA, RNA, and proteins, in the regulation of gene expression should be considered. Based on the different methods of gene regulation by lncRNAs, lncRNAs are divided into guides, scaffolds, signaling molecules, decoys, and miRNA sponges, which affect the pretranscription, transcription, and posttranscriptional levels of gene expression [34, 35]. It is now evident that silencing G4 elements in the core promoter region of oncogenes such as *KRAS* is a highly valuable and new molecular target in the treatment of cancer [36]. Some innovative approaches have suggested that lncRNAs containing G4 structures as molecular decoys for G4-binding proteins prevent G4 formation in the promotor region of oncogenes, which leads to gene transcription [37]. Therefore, the determination of whether lncRNAs inhibit G4 element formation in the promotor region of *KRAS* reveals other mechanisms by which lncRNAs regulate *KRAS* expression at the pretranscription level. The results of another study demonstrated that KRASIM, a highly conserved microprotein encoded by the putative lncRNA NCBP2-AS2, plays a tumor-suppressive role by interacting with KRAS in HCC cells. KRASIM, as the first KRAS-binding protein encoded by a lncRNA, suppresses the protein level of KRAS and inhibits the ERK signaling pathway. Therefore, sequestration of the KRAS protein with peptides encoded by lncRNAs can be considered as an alternative lncRNA-associated posttranscriptional regulatory mechanism [38].

While lncRNAs have the capacity to regulate *KRAS* expression, abnormal levels of KRAS, one of the mediators of many cellular signaling pathways, reciprocally cause diverse molecular alterations, such as dysregulation of lncRNA expression. *KRAS* amplification has been shown to be a secondary means of KRAS activation, leading to its overexpression and neoplastic transformation. It was found that the levels of a KRAS-responsive lncRNA called KIMAT1 correlate with the KRAS levels and play a positive role in maintaining tumorigenesis [39]. Another study revealed that oncogenic RAS-induced lncRNA 1 (Orilnc1) can be regulated by the RAS-RAF-MEK-ERK pathway and is required for cell proliferation in RAS/BRAF-dependent human cancers [40].

The diversity of miRNAs with their various MREs provides a greater possibility for communication between different miRNAs and ceRNAs, two irreplaceable contributors to the sponging effect. This hypothesis suggests that the sponging effect is a key molecular mechanism underlying the networks corresponding to miRNAs, oncogenic lncRNAs, and many related oncogenic drivers that control various cancer-related biochemical processes. While *KRAS*-associated miRNAs have been widely studied in cancer, the role of *KRAS*-related lncRNAs in promoting cancer progression needs to be carefully examined. The ever-increasing number of *KRAS*-specific lncRNAs strongly indicates their potential contribution to and critical roles in the entire process of *KRAS*-driven carcinogenesis. This review compiles the current knowledge of *KRAS*-related oncogenic lncRNAs by considering their aberrant expression and their mechanism of action through sponging effects on *KRAS*-targeting miRNAs.

## Noncoding RNAs in KRAS-driven cancers

The noncoding transcriptome consists of a variety of different RNA types, such as transfer RNA (tRNAs), ribosomal RNAs (rRNAs), small nuclear RNAs (snRNAs), small nucleolar RNAs (snoRNAs), circular RNAs (circRNAs), miRNAs, and lncRNAs. Other than miRNAs and lncRNAs, as noncoding RNAs that play roles in tumorigenesis, accumulating evidence indicates that altered processing or activity of other RNA species can similarly contribute to cancer [13]. Intact tRNAs and tRNA fragments (tRFs) are correlated with tumorigenesis [41]. Upregulation of specific tRNA expression in breast cancers by the enhancement of the translation of specific transcripts has been demonstrated in the progression of metastasis [42]. In particular, a proportion of tRFs that are of the same size as miRNAs and associated with Argonaute are able to function as miRNAs. To confirm the oncogenic activity of tRFs, altered levels have been indicated in leukemia and solid cancers [42–44]. It has been reported that some tRNA fragments, such as ts-47s and ts-46s, are upregulated by KRAS and PIK3CA mutations, respectively, leading to breast cancer chemoresistance [45, 46]. The results indicated that the expression of tRFs can be influenced by oncogenic mutations with a possible role in the promotion of carcinogenic processes. Other findings have demonstrated that the expression of different tRNAs corresponds to differences in KRAS protein levels. This proved that some translational programs, such as overexpression of proliferative tRNAs, have the ability to enhance the protein synthesis of oncogenes, including KRAS [47].

A wide range of data has indicated the fundamental importance of ribosomal biogenesis and its relationship with cell proliferation in many aspects of malignant transformations [48]. A series of rare inherited disorders leading to the production of altered ribosomes (so-called ribosomopathies) have even been characterized by a strong risk of cancer onset [49]. An imbalance in the ribosome biogenesis rate via an increase in ribosomal DNA transcription or an alteration in mature rRNA or ribosomal protein production may ultimately lead to the inactivation of p53 through different mechanisms [50]. As a consequence of *p53* repression, acquisition of cellular phenotypic changes characteristic of epithelial-mesenchymal transition (EMT) results in increased cell invasiveness. In addition, it has been reported that nuclear epithelial cell transforming sequence 2 (ECT2) with GEF activity is required for *KRAS-p53* lung tumorigenesis in vivo. ECT2-dependent ribosomal DNA transcription and activation of rRNA synthesis ultimately lead to neoplastic transformation [51]. In addition, nuclear and nucleolar superoxide dismutase are essential for lung cancer cell proliferation through interaction with the PeBoW complex and regulation of pre-rRNA maturation [52].

The RNA components of the spliceosome, uridine-rich (U) snRNAs, can regulate tissue-specific and cancer-specific alternative splicing [53]. Notably, recurrent mutations in U1 snRNA, as one of the most abundant noncoding RNAs, have been recently identified in multiple cancer types and play an important role in the splicing of pre-mRNAs [54]. Collectively, these studies indicate that abnormalities in U1 snRNA and alternative splicing of pre-mRNA are emerging as potentially important drivers of cancer [54, 55]. An alternative mechanism underlying changes in the U1 levels in alternations of cancer gene expression is changes in 3’-untranslated region (UTR) length, leading to the removal of miRNA binding sites. U1 overexpression lengthens the 3ʹUTR of KRAS to include a miRNA let-7 binding site with tumor-suppressive activity [56].

snoRNAs are conserved noncoding RNAs responsible for ribonucleoprotein guidance in cells for RNA posttranscriptional modification [57]. A study on the characterization of small snoRNAs in cancer identified an unexpected role for specific snoRNAs in the modulation of KRAS-driven carcinogenesis [58]. A human protein microarray screen discovered SNORD50A and SNORD50B as two snoRNAs that bind to KRAS. The results showed that loss of SNORD50A and SNORD50B expression enhances the amount of GTP-bound and active KRAS, leading to hyperactivated RAS-ERK1/ERK2 signaling [58]. The soluble NSF attachment protein receptor (SNARE) protein superfamily, which is critical for membrane fusion, is responsible for the vesicular transport that is essential for KRAS trafficking to the plasma membrane and active signaling [59]. In 2019, Che et al. found that the SNORD50A/B snoRNAs, as antagonists of SNAP23, SNAP29, and VAMP3 SNARE proteins, inhibit the process of KRAS localization to the membrane [59].

circRNAs constitute a distinct type of endogenous abundant noncoding RNA with a closed-loop structure and have been found to be overexpressed in cancers [60]. Strikingly, similar to lncRNAs, circRNAs have the potential to act as oncogenes or tumor suppressors, possibly by acting as sponges for miRNAs. Gorospe et al. found that circPVT1, as a circRNA, regulated the availability of let-7 miRNA, a well-characterized tumor suppressor with a target site on KRAS mRNA. This suggests that circPVT1, whose expression is elevated in dividing cells and downregulated in senescent cells, can be considered a KRAS-related circRNA that acts by sponging let-7 [61]. Other results showed that a circRNA derived from Golgi glycoprotein 1 mRNA regulates *KRAS* expression and then promotes colorectal cancer development by targeting miR-622 [62].

Many studies have presented remarkable details of systematic alterations in the form of noncoding RNAs, such as miRNAs, lncRNAs, snRNAs, and circRNAs, with impacts on multiple facets of tumorigenesis.

## KRAS-related lncRNAs in solid tumors

Aberrant regulation of oncogenes, tumor suppressor genes, and miRNA genes are crucial in the pathogenesis of cancer. These alterations are sequential multistep processes that can ultimately contribute to malignant transformation [63]. The crucial roles of miRNAs in various biological processes, such as cell proliferation, tumor initiation, EMT, and tumor invasion, are directly related to malignancy [64]. Several studies have identified many tumor suppressor miRNAs targeting the *KRAS* oncogene in human cancers, which affect cancer-associated cellular and molecular mechanisms [65, 66]. Notably, research progress on the interactions between lncRNAs and miRNAs in human cancer has introduced an extra layer of complexity in the miRNA-target interaction network [31]. With the development of the analysis of regulatory networks, differential expression, and signaling pathways, lncRNAs have emerged as crucial regulators in various biological processes [67, 68].

**Fig. 2:**
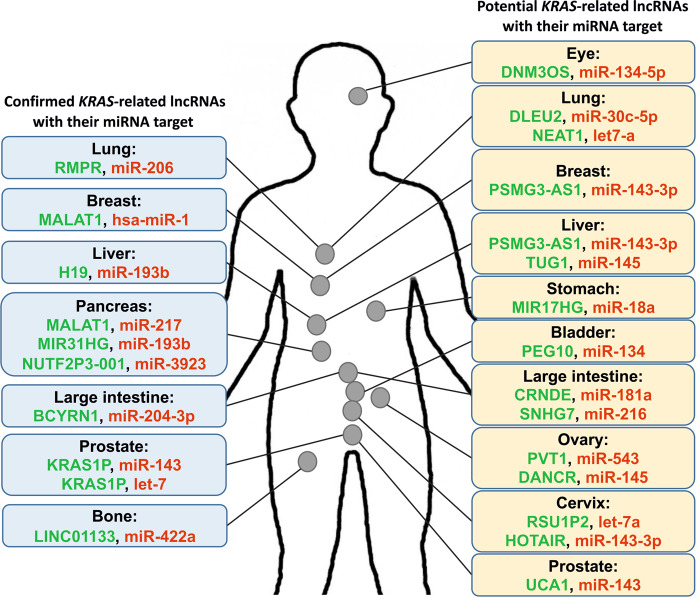
Lists of the confirmed (left) and potential (right) KRAS-related lncRNAs in different tissues. lncRNAs are presented in green, indicating their upregulation as oncogenic regulators in carcinogenesis. miRNAs with tumor suppressor activity are presented in red, indicating their repression due to the sponging effect of lncRNAs in malignancies. The left and right panels illustrate the confirmed and potential KRAS-related lncRNAs as well as their miRNAs, respectively (Supplementary Table S1).

In this review, we mainly focus on confirmed *KRAS*-related lncRNAs whose oncogenic roles as suppressors of *KRAS*-targeting miRNAs have been verified (Fig. 2). These lncRNAs act as molecular sponges of *KRAS*-targeting miRNAs, most likely contributing to *KRAS* upregulation. We also summarize a large number of lncRNAs potentially capable of regulating KRAS, possibly through sponging of previously recognized *KRAS*-targeting miRNAs (Fig. 2) [31].

## Confirmed KRAS-related lncRNAs

### MALAT1

MALAT1, which was first identified in lung cancer, plays an important role in the pathogenesis of various human diseases, such as cancer [69–71] and autoimmune and inflammatory diseases [72]. MALAT1 behaves as an oncogene in the initiation and progression of many cancers [73, 74]. MALAT1, as a molecular sponge of miR-217, an inhibitor of *KRAS* [75], promotes *KRAS* signaling in pancreatic ductal adenocarcinoma (PDAC) [76]. In this regard, knockdown of MALAT1 results in a significant reduction in MEK and ERK1/2 phosphorylation by attenuating KRAS protein expression, emphasizing the role of MALAT1 in protecting *KRAS* mRNA from repression by miR-217 [76]. Moreover, miR-1 has been shown to suppress breast cancer development by downregulating *KRAS* and MALAT1 transcription, which emphasizes the potential role of miR-1 as a tumor-suppressive miRNA and MALAT1 as an oncogenic lncRNA via the regulation of *KRAS* [66].

### MIR31HG

MIR31HG is a lncRNA with 2166 nucleotides that originates from the intronic region of the Harbi1 gene and is responsible for coding miR-31. MIR31HG is markedly upregulated in cancer tissues, with potential roles in cancer initiation, progression, and metastasis. It was confirmed that MIR31HG facilitates esophageal squamous cell carcinoma cell proliferation and functions as a ceRNA by sponging miR-34a, allowing upregulation of *c-Met* [77]. MIR31HG inhibits oncogene-induced cell senescence by regulating transcription of the tumor suppressor p16 (*INK4A*) [78]. The interaction of the MIR31HG transcript with the genomic regions of *INK4A* and MIR31HG contributes to the recruitment of polycomb-group protein complexes and then the repression of *INK4A*. In addition, *SP1*-induced MIR31HG was found to be significantly upregulated in NSCLC tissues and cell lines, which promotes cell migration and invasion by sponging miR-214 [79]. It has been reported that miR-193b is able to directly target MIR31HG, resulting in cancer progression by counteracting miR-193b in pancreatic cancer [80]. Based on the significant role of the *KRAS* mutation in pancreatic cancer, these results confirm the potential role of MIR31HG in the malignant transformation of different tumors, including *KRAS*-driven pancreatic cancer.

### KRAS1P

KRAS1P is considered as a pseudogene of *KRAS*. Its expression is amplified in most cancers with mutated *KRAS*, which indicates a positive correlation between these genes. The transcript levels of *KRAS* and KRAS1P correlate directly in prostate cancer, neuroblastoma, retinoblastoma, and hepatocellular carcinoma (HCC), which illustrates a proto-oncogenic role of KRAS1P in cancer [81–83]. While the detailed mechanism by which KRAS1P regulates *KRAS* as a pseudogene-derived noncoding RNA has not been well recognized, its activity as a sponge for miRNAs that bind to the 3ʹUTR of *KRAS* has been proposed [84]. Two studies have reported the possible role of KRAS1P as a ceRNA with binding sites for some *KRAS*-targeting miRNAs, such as miR-143 and the let-7 miRNA family [85, 86]. Thus, KRAS1P can potentially act as an oncogenic lncRNA to inhibit degradation of the *KRAS* transcript [84].

### BCYRN1

BCYRN1 is a newly identified brain cytoplasmic lncRNA of 200 nucleotides, which is transcribed from human chromosome 2p21. The high expression of BCYRN1 in various tumor cell lines suggests the role of BCYRN1 as an oncogenic lncRNA [87, 88]. In gastric cancer tissues, it is associated with tumor depth, lymph node metastasis, cell proliferation, cell cycle progression, migration, and invasion [89]. BCYRN1 is upregulated in colorectal cancer (CRC) tissues, which is related to tumor growth and advanced pathological stages via *NPR3* overexpression [90]. Moreover, the promotion of glycolysis and tumor progression in non-small cell lung cancer (NSCLC) are observed as the result of BCYRN1 overexpression [91]. High BCYRN1 expression induces glycolysis through the repression of miR-149 and upregulation of *PKM2* as the target of miR-149. Strikingly, as a ceRNA, BCYRN1 affects the development of CRC via regulation of the miR-204-3p/*KRAS* axis [92]. Therefore, negative regulation of *KRAS* by miR-204-3p suggests BCYRN1 as another confirmed *KRAS*-related lncRNA.

### NUTF2P3-001

Overexpression of NUTF2P3-001 in pancreatic cancer and chronic pancreatitis tissues is positively correlated with cancer cell characteristics, such as tumor size and distant metastasis [93]. It was reported that NUTF2P3-001, as an oncogenic lncRNA, competes with the 3′UTR of *KRAS* mRNA for binding to miR-3923. In addition, downregulation of NUTF2P3-001 inhibits the viability, proliferation, and invasion of pancreatic cancer cells and contributes to a decrease in *KRAS* expression [93]. Hence, these data provide an alternative lncRNA-mediated regulatory mechanism for the tumor oncogene *KRAS*.

### RMRP

RMRP lncRNA is widely expressed in different human and mouse tissues [94]. Previous studies have indicated that the expression of RMRP is dysregulated in gastric cancer [95]. Suppression of miR-206 by RMRP positively modulates Cyclin D2 expression and cell cycle progression, which provides us with a better understanding of the mechanism underlying RMPR carcinogenesis [96]. Furthermore, ectopic expression of RMRP was observed to promote cell proliferation, colony formation, and invasion in lung adenocarcinoma [97]. It was indicated that miR-206 acts as a tumor suppressor miRNA in oral squamous cell carcinoma by directly targeting *KRAS* [98]. Inhibition of miR-206 by RMRP was demonstrated to result in overexpression of *KRAS*, *FMNL2*, and *SOX9* in lung adenocarcinoma [99], confirming RMPR as one of the *KRAS*-related lncRNAs.

### H19

H19, with both oncogenic and tumor suppressor activities, acts as a double-edged sword via mechanisms such as miRNA sponging [100]. The let-7 family miRNAs that control human RAS oncogene expression are often downregulated in human cancers [86, 101, 102]. H19 possesses both canonical and noncanonical binding sites for the let-7 family of miRNAs, which plays predominant roles not only in cancer but also in development and metabolism [103]. H19 promotes pancreatic cancer metastasis by inhibiting let-7 suppression on its target *HMGA2*-mediated EMT in PDACs [100, 104]. Considering the role of let-7 in targeting *KRAS*, H19 may influence *KRAS* expression levels in PDAC. To confirm other sponging effects of H19, H19 overexpression exerted proangiogenic effects, possibly by downregulating miR-181a and inducing the JNK and AMPK signaling pathways to facilitate angiogenesis [30]. Considering the tumor-suppressive effect of miR-181a via downregulation of *KRAS* and the role of the *KRAS* mutation in vascular malformations, it is assumed that H19 has an indirect effect on *KRAS* upregulation [105, 106]. This can also be mediated by miR-193b, another *KRAS*-regulating miRNA [107]. Overexpression of H19 has been shown to attenuate miR-193b-mediated inhibition of multiple driver oncogenes, including *EGFR*, *KRAS*, *PTEN*, *IGF1R*, and *MAPK1*, suggesting that lncRNA H19 serves as a *KRAS* regulator through miR-193b sponging [108].

### LINC01133

LINC01133, with a length of 1154 nucleotides, is located on chromosome 1q23.2 and was first reported to be involved in CRC and NSCLC [109, 110]. A positive correlation has been found between high LINC01133 expression and poor prognosis in patients. LINC01133 downregulation leads to the repression of proliferation and invasion of lung cancer cells [111]. Nevertheless, other studies have shown low LINC01133 expression in CRC and breast cancer tissues [112, 113]. Therefore, it can be concluded that the expression levels of LINC01133 vary among various types of cancer, suggesting that there is a tissue-specific regulation of its expression that may be directly related to its function. Other results showed that LINC01133 aggravates the proliferation, migration, and invasion of osteosarcoma by sponging miR-422a, which targets *KRAS*, exerting antitumor effects [114, 115].

### SLCO4A1-AS1

The role of SLCO4A1-AS1 in the tumorigenesis of CRC has been demonstrated in several studies, confirming its upregulation in CRC tissues and its relation with poor prognosis and tumor metastasis [116, 117]. SLCO4A1-AS1 has been reported to serve as an oncogenic lncRNA in CRC by activating the WNT/β-catenin signaling pathway [117]. The oncogenic role of SLCO4A1-AS1 in CRC promotion has been attributed to the stabilization of SLCO4A1, a transmembrane protein with sodium-independent organic anion transporter activity. In addition, the axis of the SLCO4A1-AS1/miR-508-3p/PARD3 autophagy pathway has been proposed as another carcinogenic mechanism of SLCO4A1-AS1 in the development of CRC through a sponging effect [116]. SLCO4A1-AS1 knockdown in HCT116 and SW480 cells led to the downregulation of *EGFR*, *KRAS*, *BRAF*, and *MAP3K1* expression [118]. Therefore, SLCO4A1-AS1 can be considered as a *KRAS*-related lncRNA. However, the corresponding miRNA has not yet been identified.

## Potential KRAS-related lncRNAs

On the basis of the significant role of *KRAS* oncogenic mutations, many miRNAs that target *KRAS* have been discovered in many human cancer tissues [119, 120]. The inhibitory effect of miRNAs on *KRAS* expression led us to search for miRNAs that are sponged by oncogenic lncRNAs to find potential *KRAS*-related lncRNAs. Therefore, a review of the previously recognized KRAS-targeting miRNAs helps us to predict some oncogenic lncRNAs with sponging effects, which may participate in the regulation of *KRAS*. To identify potential *KRAS*-related lncRNAs, two steps were taken. In the first step, a collection of miRNAs that target *KRAS* were identified. Second, an extensive literature study was performed to determine lncRNAs with sponging effects on the miRNAs (Fig. 2). For example, a significant role of miR-143 in the inhibition of *KRAS* translation was confirmed to contribute to the suppression of cell growth [85]. In this regard, other supporting documents showed the interaction of PSMG3-AS1 lncRNA as a sponge with miR-143-3p in HCC and breast cancer tissues [16, 121]. According to the targeting of *KRAS* by miR-143 and the sponging effect of PSMG3-AS1 on this miRNA, it can be assumed that PSMG3-AS1 can be a potential *KRAS*-associated lncRNA. Similarly, miR-181a is a known miRNA with the ability to target *KRAS* mRNA. With this information, lncRNA CRNDE, whose sponging effect on miR-181a was previously confirmed, can be considered one of the other potential *KRAS*-related lncRNAs [122]. Therefore, a thorough understanding of the plethora of tumor suppressor miRNAs contributing to *KRAS-*targeting and its downregulation provides mechanistic insight into discovering potential *KRAS*-related oncogenic lncRNAs that act as molecular sponges. Accordingly, there is a large number of potential *KRAS*-related lncRNAs sponging the *KRAS*-targeting miRNAs (Fig. 2; Supplementary Table S1).

## RAS-related lncRNAs associated with leukemia

Leukemia, as a heterogeneous group of malignant neoplasms in the hematopoietic system, is classified on the basis of its clinical behavior and histological origin. Although leukemia is a common malignant cancer of the hematopoietic system, its mechanism of pathogenesis has not been fully elucidated [123]. One of the main causes of this malignancy is related to acquired and infrequently inherited genetic alterations [124]. Moreover, epigenetic alterations, such as heritable and reversible changes, can also lead to some malignant behaviors, such as cancer relapse. For instance, as well-studied leukemia, acute myeloid leukemia (AML) is a typical consequence of these abnormalities and gene mutations [125]. In addition to these valuable efforts, an urgent need to elucidate the mechanism of cancer malignancy triggered the researchers to search for new molecular systems, including regulatory transcripts such as miRNAs and lncRNAs.

Oncogenic RAS mutations are highly prevalent in hematopoietic malignancies and are associated with poor survival [126]. While somatic mutations, such as KRAS mutations, cause a series of downstream secondary alterations in the transcriptome of cancer cells, evidence showing the role of lncRNAs in the pathophysiology of hematological malignancies has drastically increased in the last decade [127]. Therefore, understanding the role of KRAS mutations in large-scale alterations in the transcriptional profiles of leukemia cells, including the dysregulation of lncRNA expression, provides more details on the pathogenic mechanisms. In this regard, the results of a pairwise analysis study comparing patients with KRAS mutations showed 26 differentially expressed lncRNAs (17 upregulated and 9 downregulated) compared to juvenile myelomonocytic leukemia (JMML) patients without this mutation [128]. Other differentially expressed RNAs between JMML patients and normal bone marrow controls revealed that the expression of 29 (19 up- and 10 downregulated) lncRNAs was dysregulated in the subgroup of KRAS-mutant patients with overexpressed lnc-ACOT9-1 [129]. lncRNA MORRBID regulates the lifespan of short-lived myeloid cells in response to extracellular pro-survival signals through the suppression of the pro-apoptotic gene BCL2L11 (also known as BIM) [130]. The high expression of MORRBID accompanied by *KRAS* and *NRAS* mutations is associated with poor overall survival of JMML patients [131].

Although the exact mechanism by which KRAS-related lncRNAs function in leukemia has not been elucidated, the sponging effect on miRNAs can be considered one of the regulatory procedures. Wang and colleagues demonstrated the role of MALAT1 in sponging miR-101 to inhibit its interaction with the 3ʹUTR of its target mRNA, myeloid cell leukemia 1 (MCL1). This competition between MALAT1 and MCL1 causes a decrease in MCL1 expression and a consequent increase in drug resistance in lung cancer [132]. In addition to the contribution of lncRNAs in leukemogenesis, recent studies on the role of lncRNAs as biomarkers in the diagnosis, prognosis, and therapeutic response have emphasized lncRNAs as essential regulatory factors in leukemia patients [133–135].

### lncRNAs as therapeutic targets

lncRNAs are key regulators of gene expression and act through different mechanisms, including genomic imprinting, epigenetic regulation, mRNA and protein stability regulation, protein sequestration, miRNA sponging, protein translation regulation, and alternative splicing. Therefore, not only sponging effects but also other mechanisms are involved in gene regulation by lncRNAs, which provides the possible application of extensive therapeutic strategies [136].

With rapid developments in high-throughput screening methods and bioinformatics, large numbers of cancer-related genes and their associated regulatory lncRNAs will be discovered in the near future [137–139]. Considering the critical roles of lncRNAs in malignancies, lncRNA-based therapeutics may represent promising approaches in cancer treatment through novel technologies [140, 141]. Antisense oligonucleotides (ASOs), which may form a DNA-RNA structure with their target RNA through base pairing rules, could be exploited as promising tools for targeting oncogenic lncRNAs [142]. Aptamers are specific structures in the form of oligonucleotides or peptide molecules that possess the ability to bind specifically and structurally to the desired target, such as lncRNA, and prevent the interactions of the lncRNA with its corresponding targets [136]. The CRISPR/Cas9 genome editing technique, a technology for the specific DNA modification of targeted genes, has been found to be a successful approach to silence the transcription of many carcinogenic lncRNAs [143]. Although the rapid development of a new generation of gene-editing tools, such as ASOs or CRISPR/Cas9-based therapy, has already shown the feasibility of gene-editing for cancer treatment, their off-target events or unstable efficiency originating from the spatiotemporal specificity of lncRNAs should also be evaluated for further clinical applications [14]. Neutralizing targeted lncRNAs by exogenous double-stranded RNA via RNA interference (RNAi) transfection is an alternative strategy that has shown some significant results due to its specificity [144]. Despite its specificity, the RNAi method efficiency is transient due to the natural instability of RNA molecules, which necessitates solid experimental analysis to confirm the practicability of this technology [145]. In contrast to oncogenic lncRNAs, some lncRNAs with tumor suppressor activity, such as CR749391 and LET, are known to be expressed at low levels in tumors [146, 147]. Thus, induction of these lncRNAs could be a possible therapeutic approach for cancer treatment. For example, viral transfection, as the main method for plasmid transmission to the target site, could be applied to transfect exogenously synthesized tumor suppressor lncRNA plasmids into cancer cells to upregulate the expression of corresponding lncRNAs. This lncRNA-based strategy could be investigated for cancer treatment; however, solid experimental analysis is required to validate the feasibility and practicability of this strategy [14]. Aside from the fact that lncRNAs themselves could serve as possible therapeutic targets, recent documents have proven the utility of peptides/proteins encoded by lncRNAs as other potential targets [148]. lncRNAs are known as RNA molecules that do not encode proteins, but recent findings have shown that peptides/proteins encoded by lncRNAs do indeed exist and surprisingly have tumorigenic effects [148]. Therefore, peptides/proteins encoded by lncRNAs might be hidden oncopeptides/oncoproteins representing promising drug targets for treating tumor growth [148]. On the other hand, some proteins encoded by lncRNAs have tumor-suppressive effects that inhibit the carcinogenesis of oncoproteins such as KRAS [38]. Taken together, these findings suggest that lncRNAs could serve as novel therapeutic targets for cancer therapy.

## Conclusion and perspective

Approximately 25% of all human cancers have oncogenic mutations in the *RAS* family of oncogenes, most frequently the *KRAS* gene, resulting in the aberrant activation of RAS proteins and consequently their downstream pathways and leading to malignant transformation. To date, diverse therapeutic approaches have been used to interfere with mutant *KRAS*-mediated signaling. Although KRAS proto-oncogene mutations are responsible for the conversion of *KRAS* to its oncoprotein form with increased activity, suppression of mutant *KRAS* gene expression could be an approach to inhibit oncoprotein production. In this review, we focused on the sponging effect as a strategy for KRAS downregulation, considering the established roles of both miRNAs and lncRNAs. The fact that the majority of lncRNAs are expressed in a highly cell- or tissue-specific manner makes them effective therapeutic targets for cancer treatment. However, many questions remain to be addressed. How many lncRNAs are functionally and clinically relevant for KRAS-driven cancers? How can we develop systematic genomic and functional approaches to understand the role of lncRNAs in the initiation, progression, and alternative metastasis of KRAS-mutant cancers? How can we integrate patient genomic and transcriptomic data with KRAS mutations to establish a lncRNA discovery pipeline to drive preclinical studies? Finally, how does a tissue-specific expression of lncRNAs provide therapeutic candidates for tissues with a higher frequency of KRAS mutation? In addition to the questions above, the authors of this review present some suggestions for future studies concerning lncRNAs as therapeutic targets. More oncogenic lncRNAs with sponging effects on other tumor-suppressive miRNAs that target KRAS or its downstream effectors should be discovered. Proteins/peptides encoded by lncRNAs and their oncogenic or tumor-suppressing effects should be investigated. The ability to target KRAS-related oncogenic lncRNAs through various methods, such as nucleic acid-based drugs, gene-editing methods, small molecule inhibitors, miRNA mimics, catalytic degradation of lncRNAs by ribozymes, targeting lncRNA secondary and tertiary structures, and synthetic lncRNA mimics, must be studied. More importantly, further characterization of interactions between oncogenic lncRNAs and associating proteins, which form ribonucleoprotein complexes and could be involved in KRAS signaling, may lead to the identification of novel therapeutic targets and the development of new anti-KRAS drugs. Hopefully, the increased success rate of nucleic acid therapeutics provides an outstanding opportunity to discover lncRNAs as viable candidates for therapeutic targets in KRAS-dependent malignant transformation.

## Supplementary information

Table S1
