# Supplementary material for: KRAS-related long noncoding RNAs in human cancers
Source: Cancer Gene Ther. 2021 Sep 6;29(5):418–27. doi: 10.1038/s41417-021-00381-x (PMC9113938; doi:10.1038/s41417-021-00381-x)
Supplement: Supplementary file 1 — Table S1 [file 41417_2021_381_MOESM1_ESM.pdf]

Supplementary Information

KRAS-related long noncoding RNAs in human cancers

Mahsa Saliani, Amin Mirzaie-Badizi, Ali Javadmanesh, Mohammad Reza Ahmadian

Institute of Biochemistry and Molecular Biology II, Medical Faculty, Heinrich-Heine University, Düsseldorf 40225, Germany

TABLE S1. List of KRAS-related lncRNAs as molecular sponges and their target miRNAs.

Type of lncRNA	lncRNA	miRNA	Cancer tissue	References
Confirmed KRAS-related lncRNAs	RMPR	miR-206	Lung	(1)
	MALAT1	has-miR-1	Breast	(2)
	H19	miR-193b	Liver	(3)
	MALAT1	miR-217	Pancreas	(4)
	MIR31HG	miR-193b	Pancreas	(5)
	NUTF2P3-001	miR-3923	Pancreas	(6)
	BCYRN1	miR-204-3p	Large intestine	(7)
	KRAS1P	miR-143	Prostate	(8)
	KRAS1P	let-7	Prostate	(8)
	LINC01133	miR-422a	Bone	(9, 10)
Potential KRAS-related lncRNAs	DNM3OS	miR-134-5p	Eye	(11)
	DLEU2	miR-30c-5p	Lung	(12)
	NEAT1	let-7a	Lung	(13)
	PSMG3-AS1	miR-143-3p	Breast	(14)
	TUG1	miR-145	Liver	(16)
	MIR17HG	miR-18a	Stomach	(17)
	CRNDE	miR-181a	Large intestine	(18)
	SNHG7	miR-216	Large intestine	(19)
	PEG10	miR-134	Bladder	(11)
	UCA1	miR-143	Prostate	(20)
	PVT1	miR-543	Ovary	(21)
	DANCR	miR-145	Ovary	(22)
	RSU1P2	let-7a	Cervix	(23)
	HOTAIR	miR-143-3p	Cervix	(24)

References:

1. Lin F, Yao L, Xiao J, Liu D, Ni Z. MiR-206 functions as a tumor suppressor and directly targets K-Ras in human oral squamous cell carcinoma. Onco Targets Ther. 2014;7:1583-91.
2. Liu R, Li J, Lai Y, Liao Y, Liu R, Qiu W. Hsa-miR-1 suppresses breast cancer development by down-regulating K-ras and long non-coding RNA MALAT1. International journal of biological macromolecules. 2015;81:491-7.
3. Ye Y, Guo J, Xiao P, Ning J, Zhang R, Liu P, et al. Macrophages-induced long noncoding RNA H19 up-regulation triggers and activates the miR-193b/MAPK1 axis and promotes cell aggressiveness in hepatocellular carcinoma. Cancer Letters. 2020;469:310-22.
4. Liu P, Yang H, Zhang J, Peng X, Lu Z, Tong W, et al. The lncRNA MALAT1 acts as a competing endogenous RNA to regulate KRAS expression by sponging miR-217 in pancreatic ductal adenocarcinoma. Scientific Reports. 2017;7(1):5186.

5. Yang H, Liu P, Zhang J, Peng X, Lu Z, Yu S, et al. Long noncoding RNA MIR31HG exhibits oncogenic property in pancreatic ductal adenocarcinoma and is negatively regulated by miR-193b. Oncogene. 2016;35(28):3647-57.
6. Li X, Deng S-j, Zhu S, Jin Y, Cui S-p, Chen J-y, et al. Hypoxia-induced lncRNA-NUTF2P3-001 contributes to tumorigenesis of pancreatic cancer by derepressing the miR-3923/KRAS pathway. Oncotarget. 2016;7(5):6000-14.
7. Yang L, Zhang Y, Bao J, Feng J-F. Long non-coding RNA BCYRN1 exerts an oncogenic role in colorectal cancer by regulating the miR-204-3p/KRAS axis. Cancer Cell International. 2020;20(1):453.
8. Poliseno L, Salmena L, Zhang J, Carver B, Haveman WJ, Pandolfi PP. A coding-independent function of gene and pseudogene mRNAs regulates tumour biology. Nature. 2010;465(7301):1033-8.
9. Zeng HF, Qiu HY, Feng FB. Long Noncoding RNA LINC01133 Functions as an miR-422a Sponge to Aggravate the Tumorigenesis of Human Osteosarcoma. Oncology research. 2018;26(3):335-43.
10. Zhang H, He Q-Y, Wang G-C, Tong D-K, Wang R-K, Ding W-B, et al. miR-422a inhibits osteosarcoma proliferation by targeting BCL2L2 and KRAS. Biosci Rep. 2018;38(2):BSR20170339.
11. Jiang F, Qi W, Wang Y, Wang W, Fan L. lncRNA PEG10 promotes cell survival, invasion and migration by sponging miR-134 in human bladder cancer. Biomedicine & pharmacotherapy = Biomedecine & pharmacotherapie. 2019;114:108814.
12. Zhou Y, Shi H, Du Y, Zhao G, Wang X, Li Q, et al. lncRNA DLEU2 modulates cell proliferation and invasion of non-small cell lung cancer by regulating miR-30c-5p/SOX9 axis. Aging (Albany NY). 2019;11(18):7386-401.
13. Qi L, Liu F, Zhang F, Zhang S, Lv L, Bi Y, et al. lncRNA NEAT1 competes against let-7a to contribute to non-small cell lung cancer proliferation and metastasis. Biomedicine & Pharmacotherapy. 2018;103:1507-15.
14. Cui Y, Fan Y, Zhao G, Zhang Q, Bao Y, Cui Y, et al. Novel lncRNA PSMG3-AS1 functions as a miR-143-3p sponge to increase the proliferation and migration of breast cancer cells. Oncol Rep. 2020;43(1):229-39.
15. Zhang J, Huang J, Chen W, Hu Z, Wang X. miR-143-3p Targets lncRNA PSMG3-AS1 to Inhibit the Proliferation of Hepatocellular Carcinoma Cells. Cancer Manag Res. 2020;12:6303.
16. Zeng B, Ye H, Chen J, Cheng D, Cai C, Chen G, et al. LncRNA TUG1 sponges miR-145 to promote cancer progression and regulate glutamine metabolism via Sirt3/GDH axis. Oncotarget. 2017;8(69):113650-61.
17. Yuan J, Tan L, Yin Z, Zhu W, Tao K, Wang G, et al. MIR17HG-miR-18a/19a axis, regulated by interferon regulatory factor-1, promotes gastric cancer metastasis via Wnt/β-catenin signalling. Cell Death & Disease. 2019;10(6):454.
18. Han P, Li J-W, Zhang B-M, Lv J-C, Li Y-M, Gu X-Y, et al. The lncRNA CRNDE promotes colorectal cancer cell proliferation and chemoresistance via miR-181a-5p-mediated regulation of Wnt/β-catenin signaling. Molecular cancer. 2017;16(1):9-.
19. Shan Y, Ma J, Pan Y, Hu J, Liu B, Jia L. LncRNA SNHG7 sponges miR-216b to promote proliferation and liver metastasis of colorectal cancer through upregulating GALNT1. Cell Death & Disease. 2018;9(7):722.
20. Yu Y, Gao F, He Q, Li G, Ding G. lncRNA UCA1 Functions as a ceRNA to Promote Prostate Cancer Progression via Sponging miR143. Molecular Therapy - Nucleic Acids. 2020;19:751-8.
21. Qu C, Dai C, Guo Y, Qin R, Liu J. Long non-coding RNA PVT1-mediated miR-543/SERPINI1 axis plays a key role in the regulatory mechanism of ovarian cancer. Biosci Rep. 2020;40(6).
22. Lin X, Yang F, Qi X, Li Q, Wang D, Yi T, et al. LncRNA DANCR promotes tumor growth and angiogenesis in ovarian cancer through direct targeting of miR-145. Mol Carcinog. 2019;58(12):2286-96.
23. Liu Q, Guo X, Que S, Yang X, Fan H, Liu M, et al. LncRNA RSU1P2 contributes to tumorigenesis by acting as a ceRNA against let-7a in cervical cancer cells. Oncotarget. 2017;8(27):43768-81.
24. Liu M, Jia J, Wang X, Liu Y, Wang C, Fan R. Long non-coding RNA HOTAIR promotes cervical cancer progression through regulating BCL2 via targeting miR-143-3p. Cancer Biol Ther. 2018;19(5):391-9.
